# Supplementary material for: Discovery of Novel Cyclic Ethers with Synergistic Antiplasmodial Activity in Combination with Valinomycin
Source: Molecules. 2021 Dec 10;26(24):7494. doi: 10.3390/molecules26247494 (PMC8708982; doi:10.3390/molecules26247494)
Supplement: Supplementary file 1 [file molecules-26-07494-s001.zip › molecules-1467964-proofed supplementary.pdf]

molecules

MDPI

Supplementary Materials:

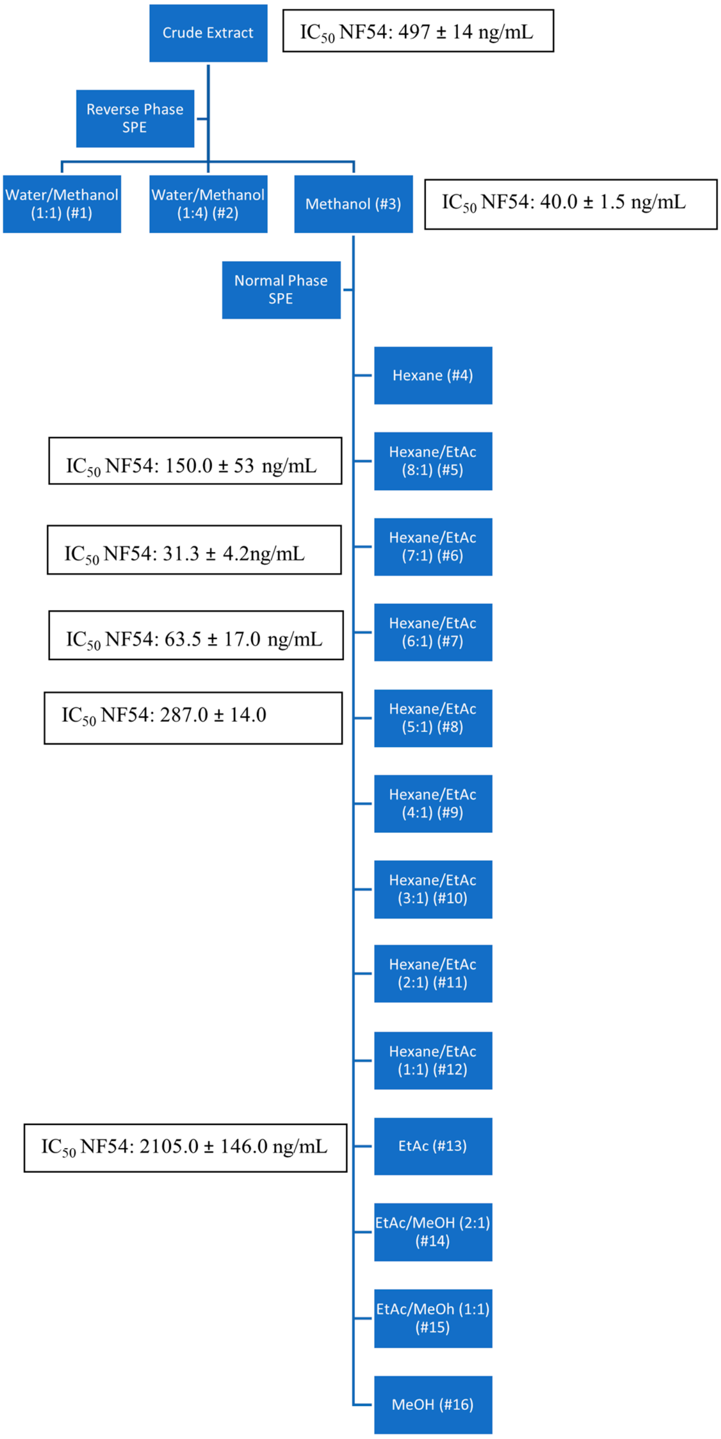

Supplementary Figure S1. Flow diagram of fractionation of *Streptomyces* strain PR3 crude extract by solid phase extraction. Each fraction is marked with its elution solvent and fraction number. Active fractions are shown with their corresponding mean antiplasmodial activity against *P. falciparum*, NF54.

Supplementary Table S1. Mass spectrometric data of the cyclodepsipeptides identified in fractions #5-#8.

Experimental Mass (Da)	*m*/*z* ($M+NH_4^+$)	Retention Time (minutes)
740.4218 (Montanastatin)	758.4552	10.21
1054.5671	1072.5997	10.27
1068.5820	1086.6151	10.33
1082.5984	1100.6308	10.46
1096.6130	1114.6461	10.56
1110.6339 (Valinomycin)	1128.6616	10.72
1124.6454	1142.6778	10.82
1138.6759	1156.6997	10.95

Supplementary Table S2. Mass spectrometric data of the cyclic propylene glycols discovered in fraction #13.

**Mass/Charge [$M+NH_4^+$] (*m*/*z*)**	**Experimental Mass (Da)**	**Theoretical Mass (Da)**	**Mass Error (ppm)**	**Chemical Formula**
772.5780	754.5437	754.5442	-0.7	$C_{39}H_{78}O_{13}$
830.6207	812.5864	812.5869	0.5	$C_{42}H_{84}O_{14}$
888.6597	870.6271	870.6274	-0.3	$C_{45}H_{90}O_{15}$
946.7076	928.6733	928.6698	3.8	$C_{48}H_{96}O_{16}$
1004.7451	986.7108	986.7116	-0.8	$C_{51}H_{102}O_{17}$
1062.7879	1044.7536	1044.7537	0.1	$C_{54}H_{108}O_{18}$
1120.8329	1102.7986	1102.7955	2.9	$C_{57}H_{114}O_{19}$
1178.8710	1160.8367	1160.8371	-0.4	$C_{60}H_{120}O_{20}$
1236.9079	1218.8736	1218.8791	-4.5	$C_{63}H_{126}O_{21}$
1294.9436	1276.9136	1276.9209	-5.7	$C_{66}H_{132}O_{22}$

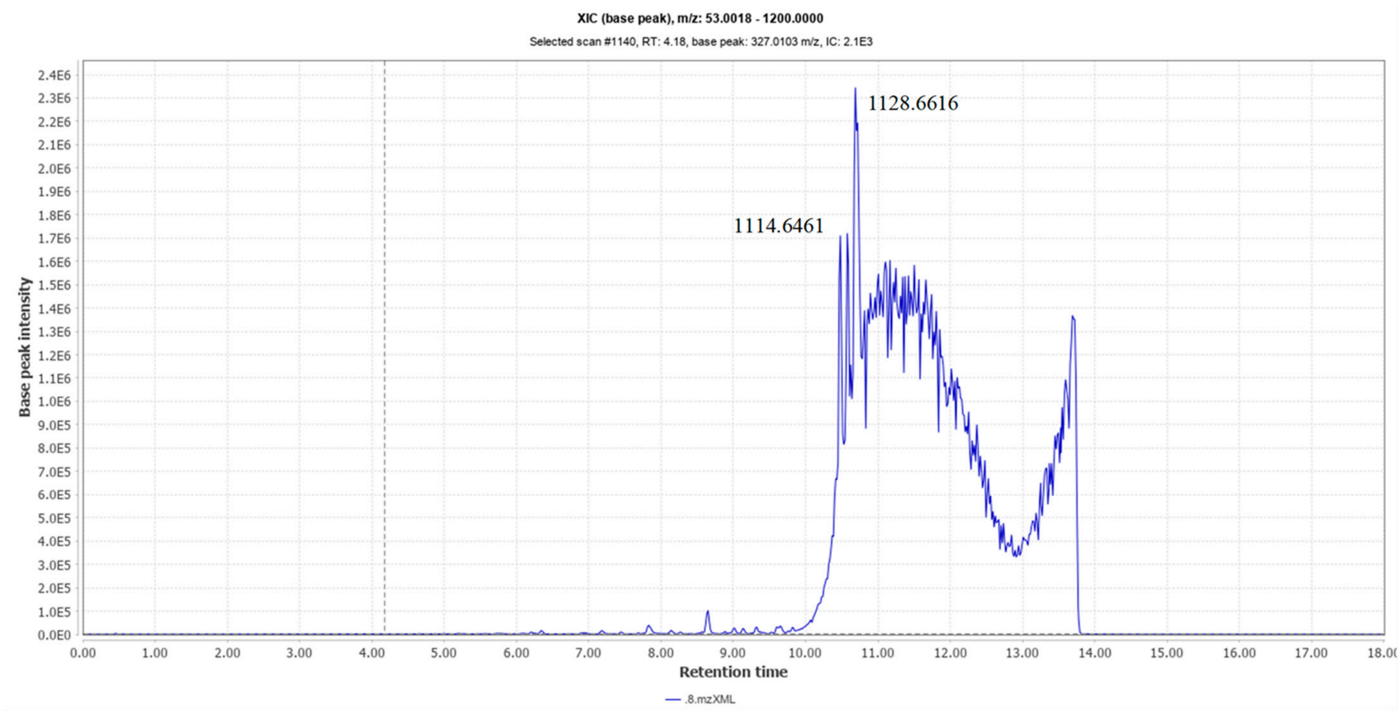

Supplementary Figure S2. Chromatogram of fraction #5 displaying the cyclodepsipeptides with the greatest intensity.

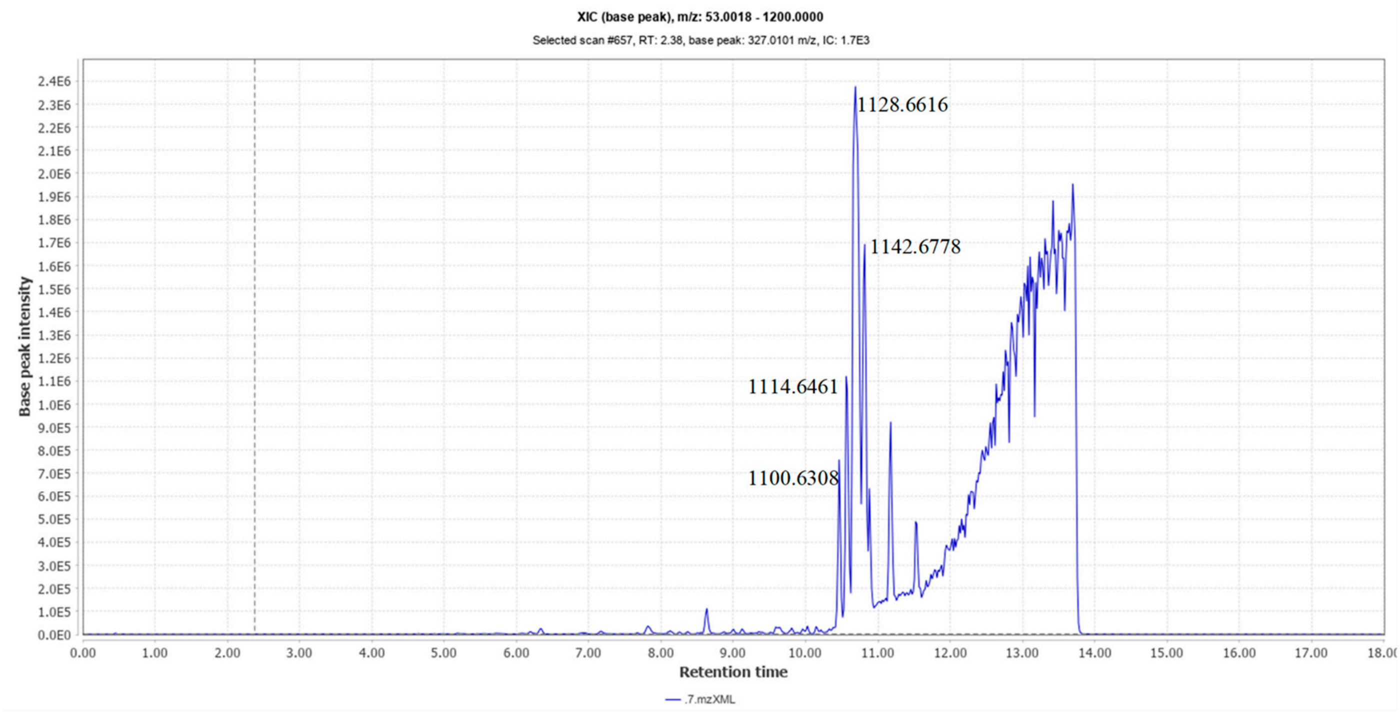

Supplementary Figure S3. Chromatogram of fraction #6 displaying the cyclodepsipeptides with the greatest intensity.

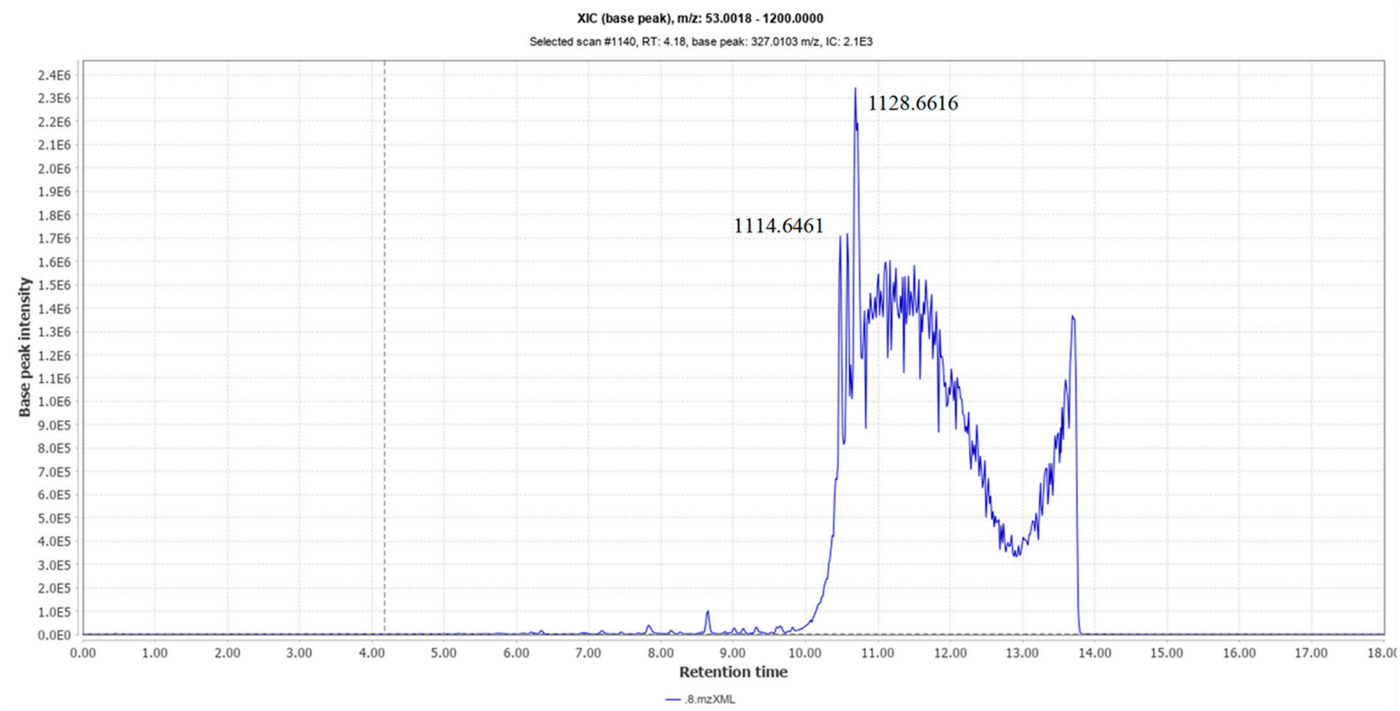

Supplementary Figure S4. Chromatogram of fraction #7 displaying the cyclodepsipeptides with the greatest intensity.

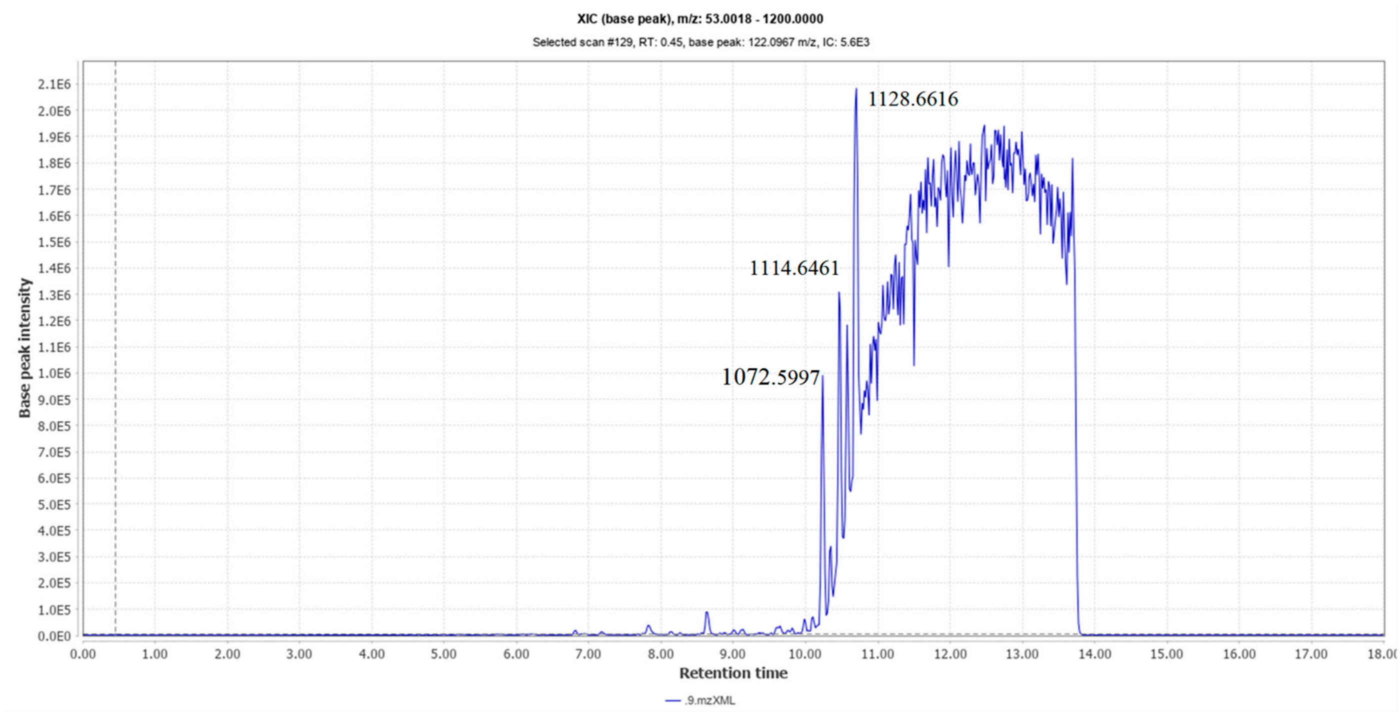

Supplementary Figure S5. Chromatogram of fraction #8 displaying the cyclodepsipeptides with the greatest intensity.

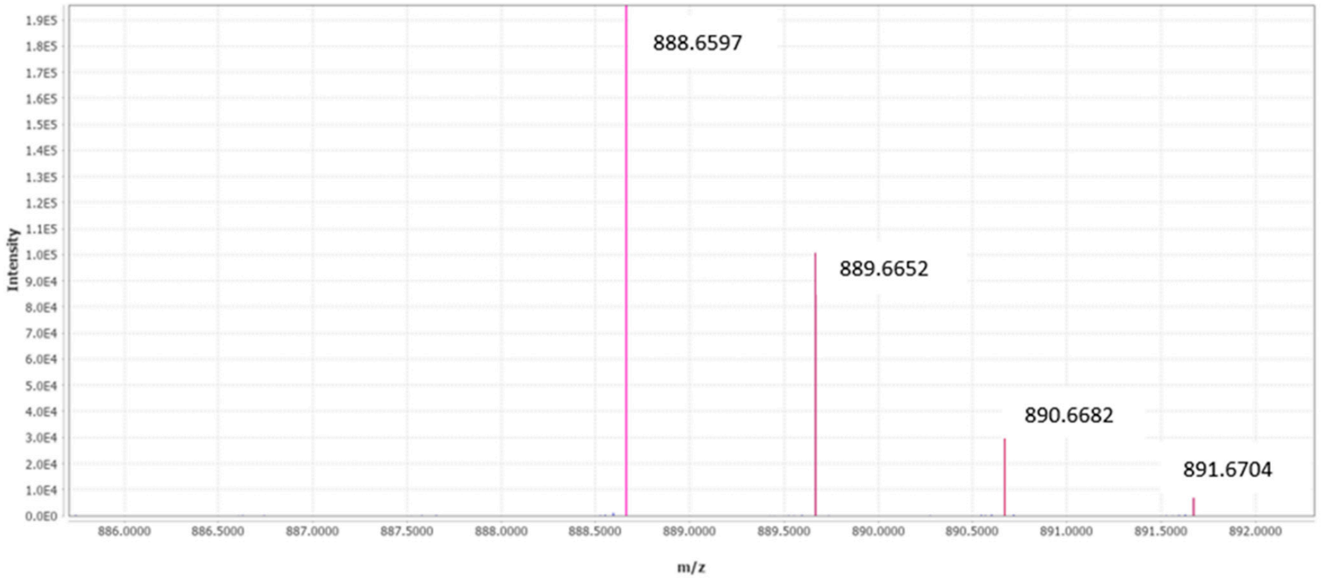

Supplementary Figure S6. Isotope pattern of compound 870 $[M+NH_4]^+$.

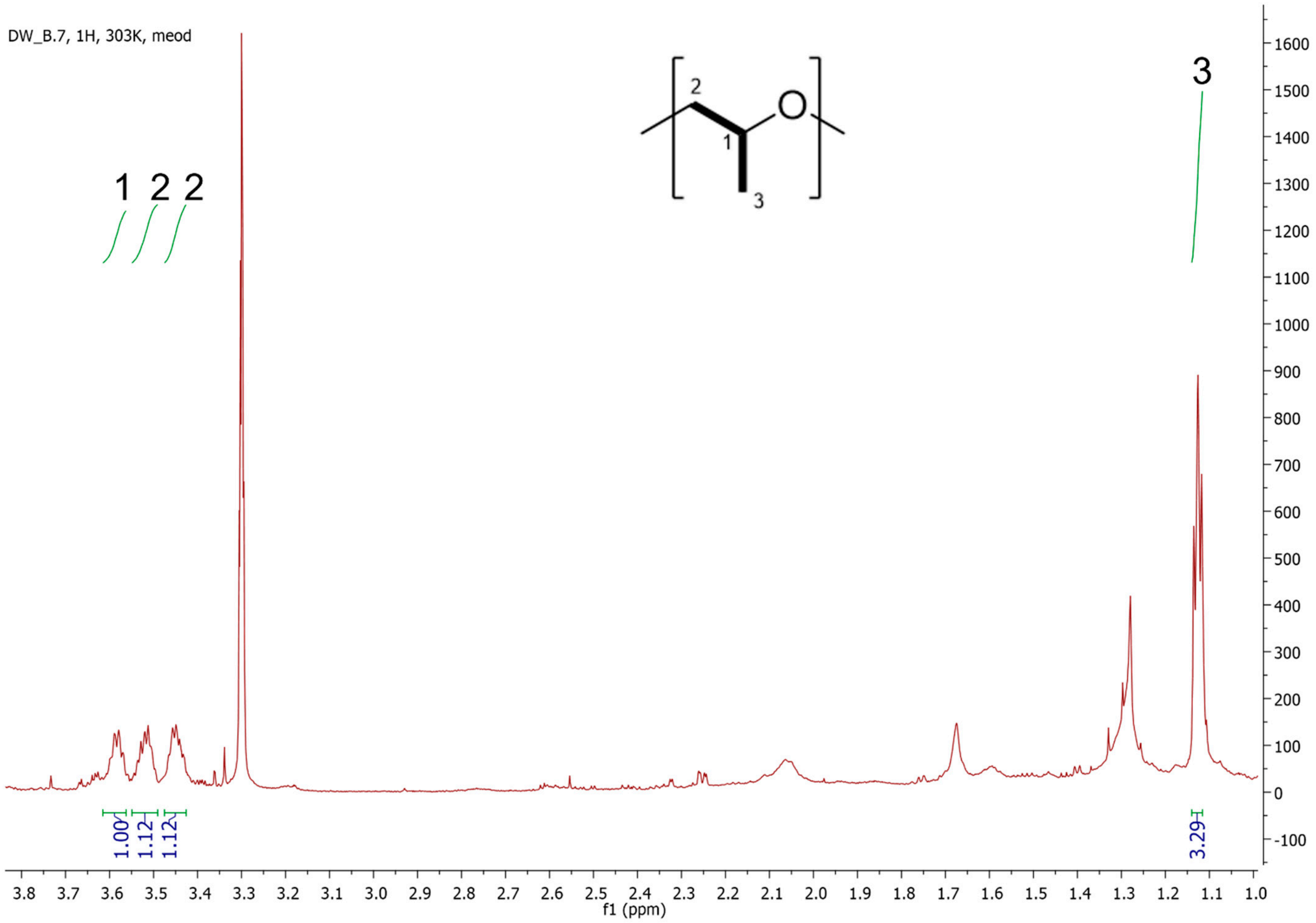

Supplementary Figure S7. ^{1}H NMR spectrum of the cPPGs (fraction #13) with the annotated propylene glycol monomer.

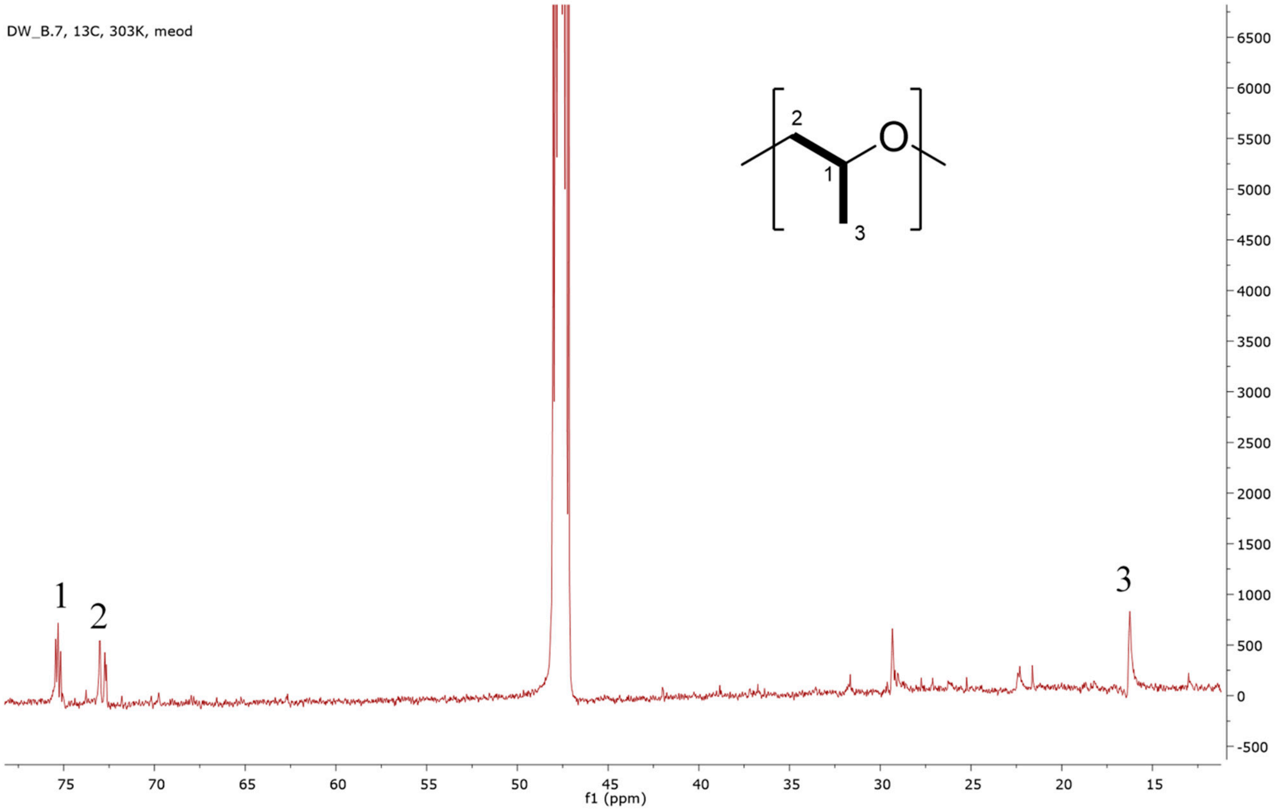

Supplementary Figure S8. ^{13}C NMR spectrum of the cPPGs (fraction #13) with the annotated propylene glycol monomer.

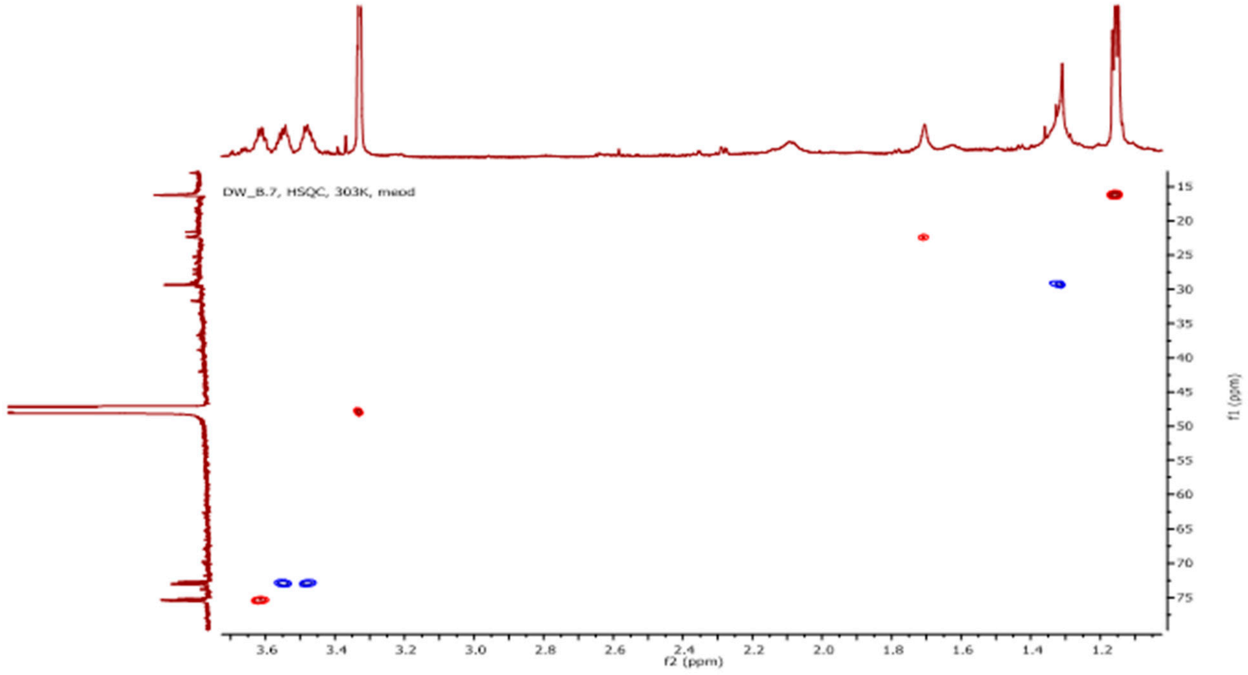

Supplementary Figure S9. HSQC spectrum of the cPPGs (fraction #13).

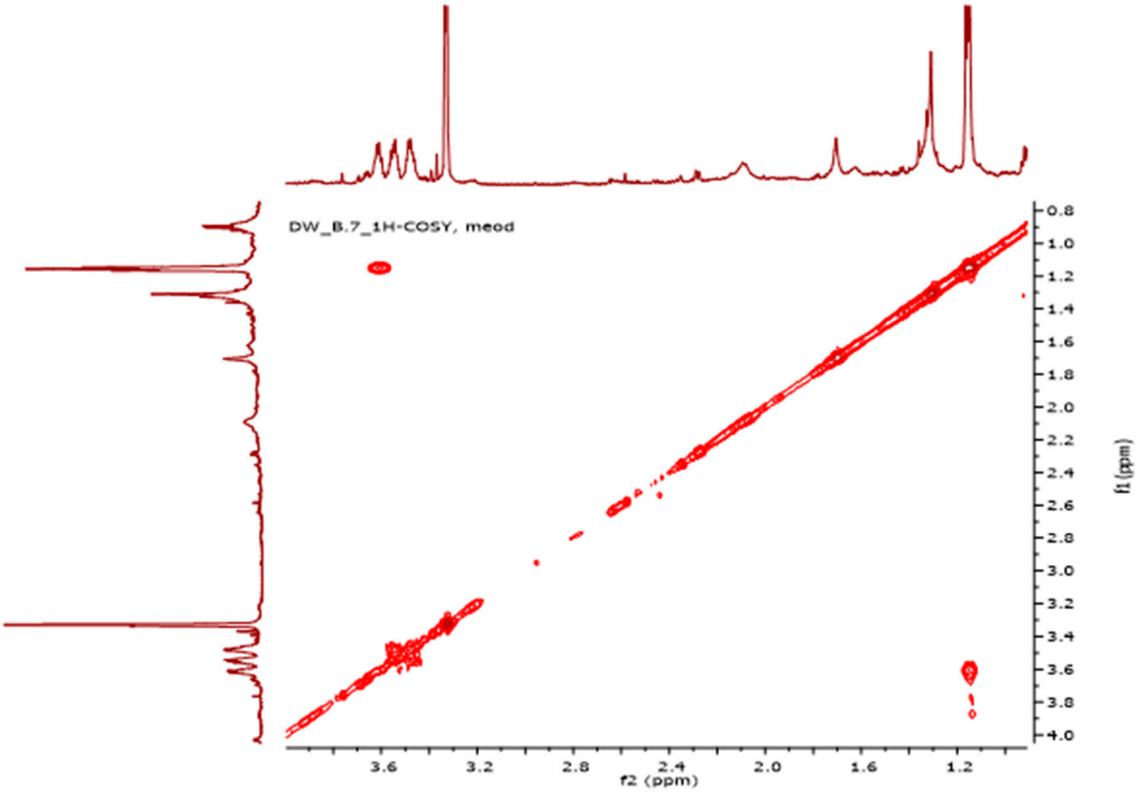

Supplementary Figure S10. ^{1}H-COSY spectrum of the cPPGs (fraction #13).

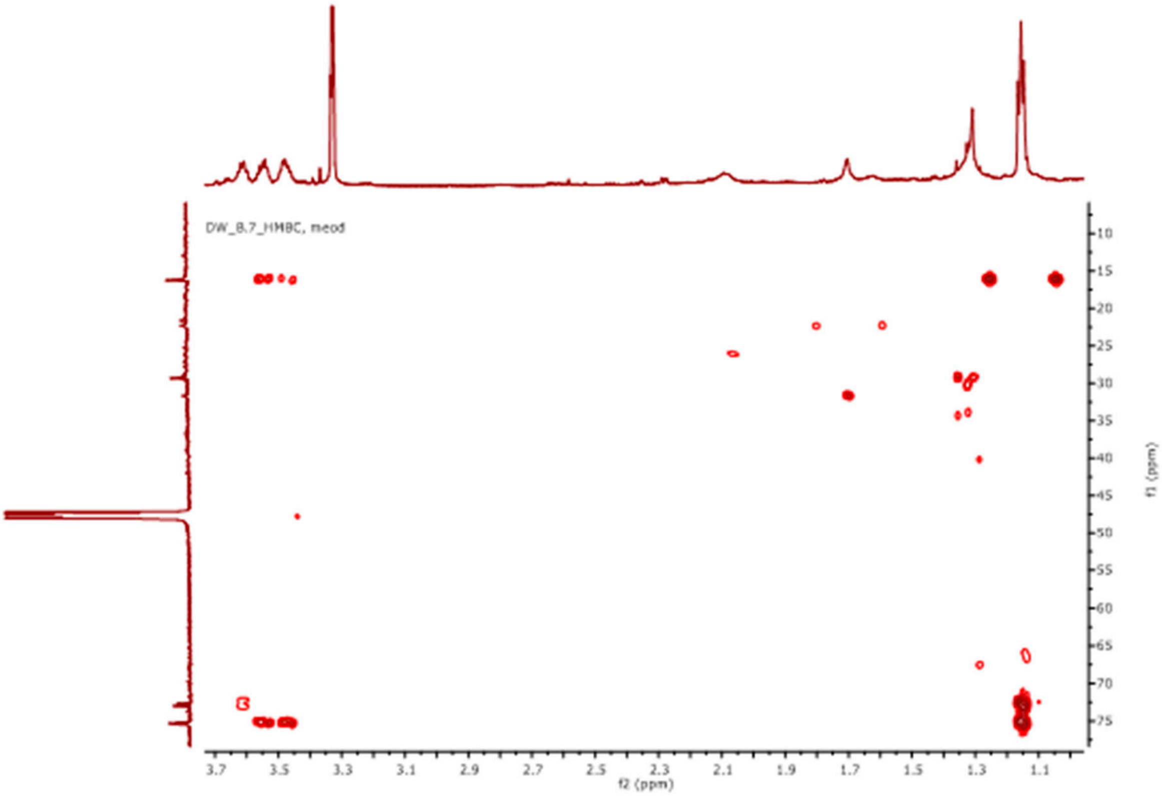

Supplementary Figure S11. HMBC spectrum of the cPPGs (fraction #13).

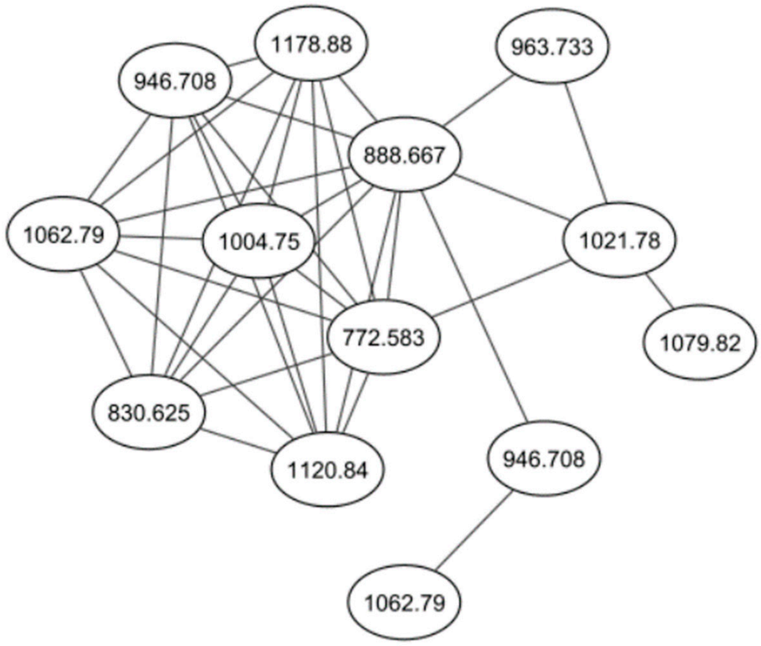

Supplementary Figure S12. Molecular network of the cPPGs found in the EtAc and water wash of the XAD-16N resin.

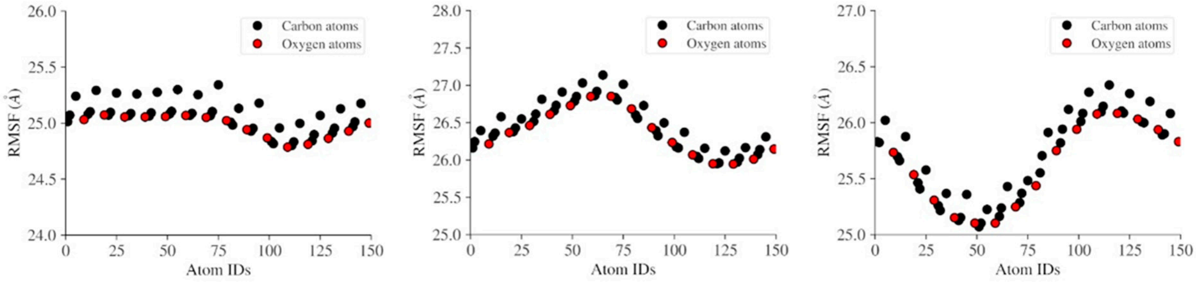

Supplementary Figure S13. RMSF's of the heavy atoms (Carbon and Oxygen) of compound 870 in varying systems (sodium, potassium and ammonium chloride solutions).

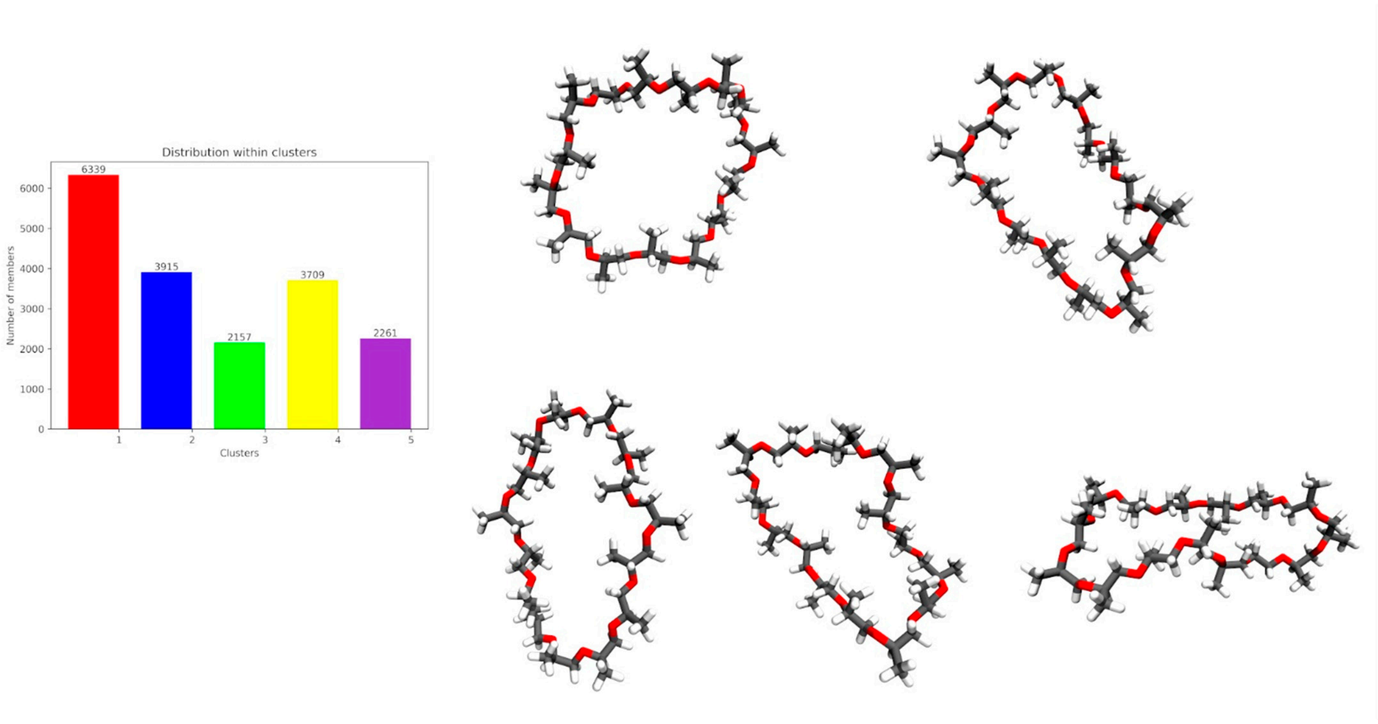

Supplementary Figure S14. Distribution of clusters found using TTClust, for compound 870 in NaCl solution.

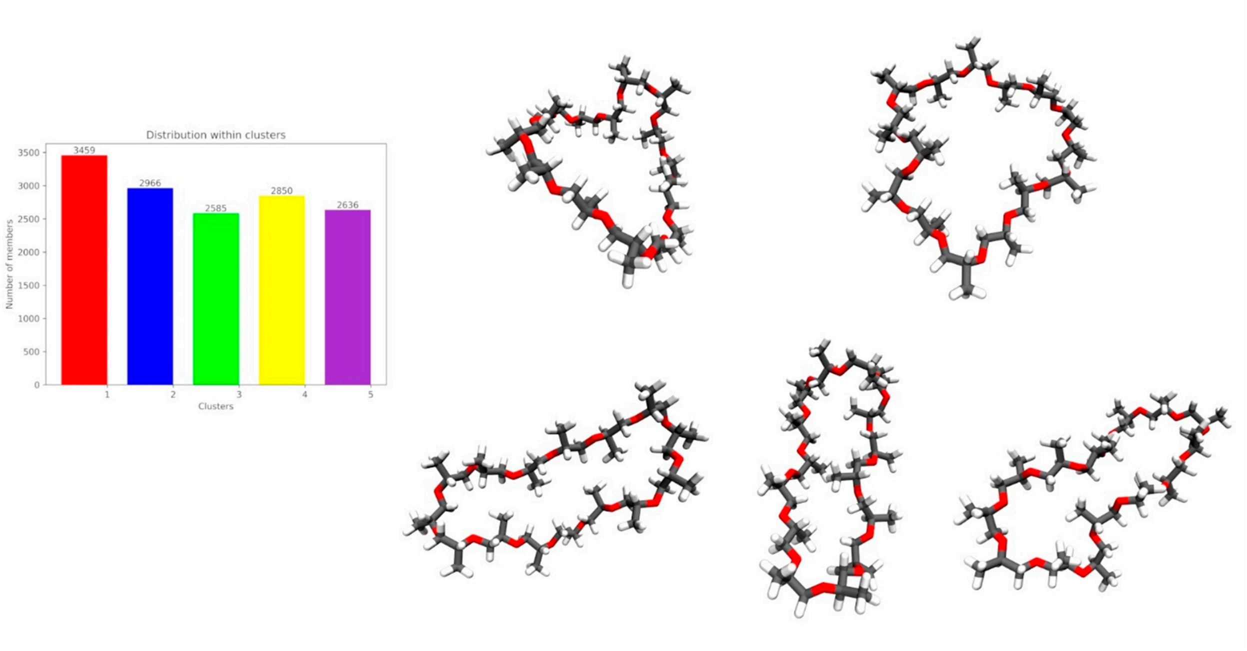

Supplementary Figure S15. Distribution of clusters found using TTClust, for compound 870 in KCl solution.

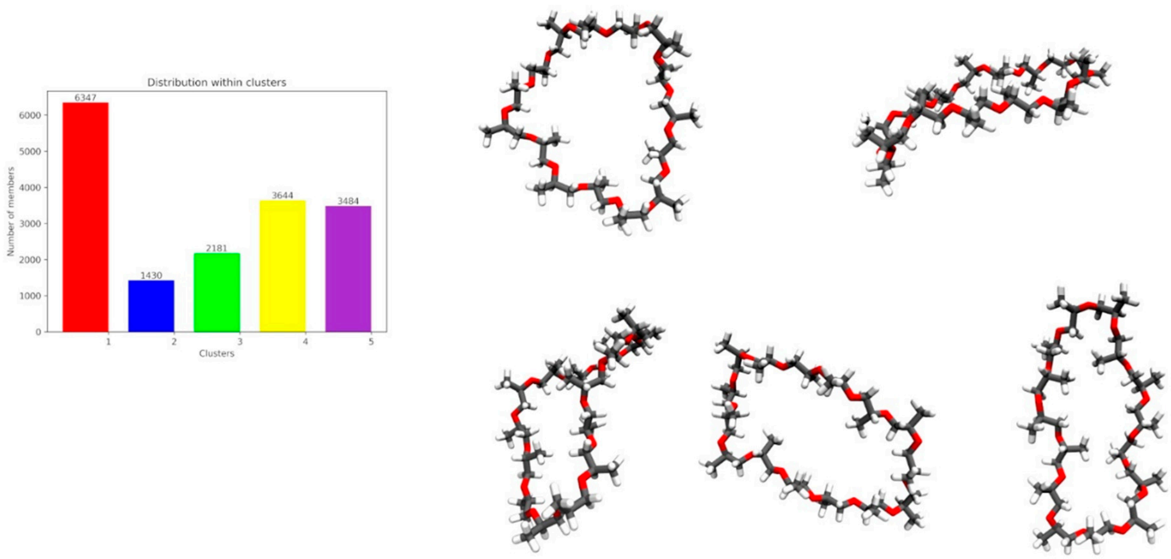

Supplementary Figure S16. Distribution of clusters found using TTClust, for compound 870 in NH_4Cl solution.

Supplementary Table S3. Mean antiplasmodial activity and fractional IC_{50}s of the cyclodepsipeptides and cyclic polypropylene glycols at each fixed-ratio and their corresponding sum of the fractional IC_{50}, N = 2 biological repeats with 4 technical repeats. The positive controls chloroquine and artesunate were also included and their activities were within acceptable ranges.

Ratio	Fraction 6 (Cyclodepsipeptides)		Fraction 13 (Cyclic Polypropylene Glycols)		Sum of Fractional IC_{50}($\sum FIC_{50}$)
	Antiplasmodial activity against *P. falciparum*, NF54 IC50 (ng/mL)	Fractional IC_{50}(FIC_{50})	Antiplasmodial activity against *P. falciparum*, NF54 IC50 (ng/mL)	Fractional IC_{50}(FIC_{50})	
5:0	23.4 ± 2.3	1.0	N/A	N/A	1.0
4:1	18.3 ± 0.4	0.78	104 ± 31.5	0.05	0.83
3:2	13.9 ± 1.3	0.60	300 ± 7.8	0.16	0.76
2:3	6.9 ± 0.7	0.30	271 ± 47.3	0.14	0.44
1:4	8.2 ± 0.7	0.35	822 ± 69.3	0.43	0.78
0:5	N/A	N/A	1910 ± 42.3	1.0	1.0
Positive Controls					
Chloroquine		6.4 ± 0.5			
Artesunate		1.8 ± 0.1			

Supplementary Table S4. Mean antiplasmodial activity and fractional IC_{50}s of valinomycin and cyclic polypropylene glycols (fraction #13) at each fixed-ratio and their corresponding sum of the fractional IC_{50}, N=2 biological repeats with 4 technical repeats. The positive controls chloroquine and artesunate were also included and their activities were within acceptable ranges.

Ratio	Valinomycin		Fraction 13 (Cyclic Polypropylene Glycols)		Sum of Fractional IC_{50}($\sum FIC_{50}$)
	Antiplasmodial activity against *P. falciparum*, NF54 IC50 (ng/mL)	Fractional IC_{50}(FIC_{50})	Antiplasmodial activity against *P. falciparum*, NF54 IC50 (ng/mL)	Fractional IC_{50}(FIC_{50})	
5:0	3.75 ± 0.77	1.000	N/A	N/A	1.000
4:1	1.86 ± 0.05	0.496	128 ± 8.83	0.072	0.57
3:2	0.90 ± 0.20	0.246	216 ± 43.5	0.121	0.37
2:3	0.75 ± 0.08	0.199	407 ± 149	0.227	0.43
1:4	0.53 ± 0.10	0.141	508.5 ± 77.0	0.376	0.51
0:5	N/A	N/A	1792 ± 547	1.000	1.000
Positive Controls					
Chloroquine	5.4 ± 1.4				
Artesunate	4.1 ± 1.3				
